# Supplementary material for: Imaging genetics of language network functional connectivity reveals links with language-related abilities, dyslexia and handedness
Source: Commun Biol. 2024 Sep 28;7:1209. doi: 10.1038/s42003-024-06890-3 (PMC11438961; doi:10.1038/s42003-024-06890-3)
Supplement: Supplementary file 1 — Supplementary Information [file 42003_2024_6890_MOESM1_ESM.pdf]

Supplementary Information Amelink et al. 2024

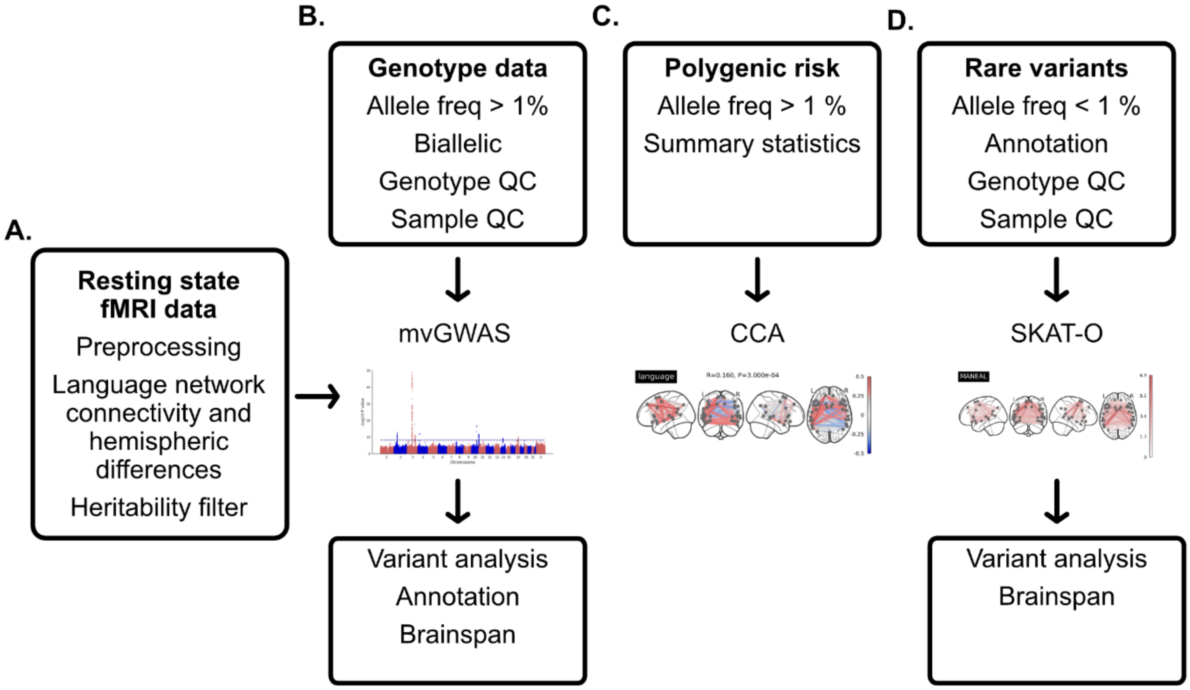

Supplementary Figure 1 - Abbreviated overview of the analysis pipelines used. **A.** We derived connectivity values and their hemispheric differences from resting state connectivity from the SENSAAS atlas that was previously developed based on several language tasks (see Introduction), filtered for heritability, and then applied three different genetic analyses to both these phenotype sets. **B.** The first analysis was a multivariate genome-wide association study (mvGWAS) based on common variant genotype data, which was annotated using FUMA, MAGMA and Brainspan data. **C.** The second analysis involved deriving of polygenic scores based on large-scale GWAS summary statistics for three phenotypes of interest: language-related performance, dyslexia, and left-handedness. We then used canonical correlation analysis (CCA) in combination with a permutation test to test the multivariate association patterns between these scores and our language network connectivity and hemispheric differences. **D.** The third analysis was an exome-wide scan using a gene-based SKAT-O test (an optimized sequence kernel association test), which was followed-up with variant association testing and annotation using Brainspan data. Allele freq represents allele frequency. Quality control is abbreviated as QC.

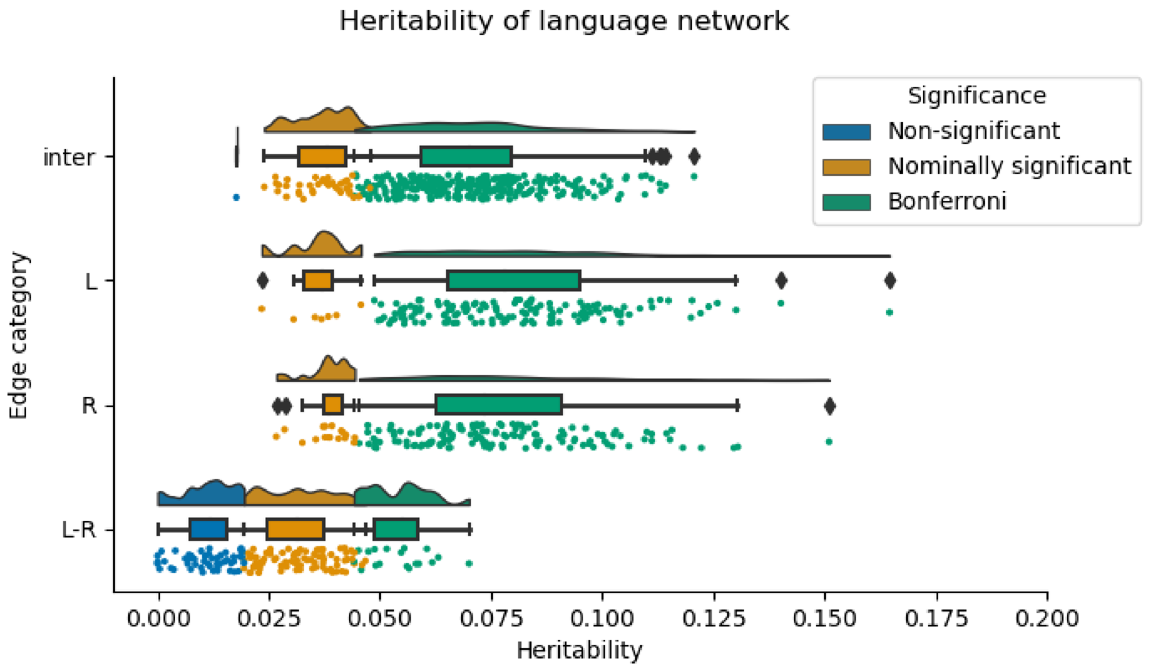

Supplementary Figure 2 – Heritability estimates from GCTA for all derived phenotypes. All non-significant phenotypes (blue) were omitted from all further analyses.

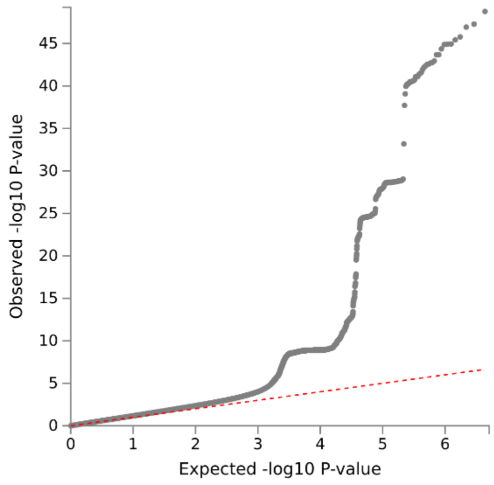

Supplementary Figure 3 – QQ plot for mvGWAS results for language network

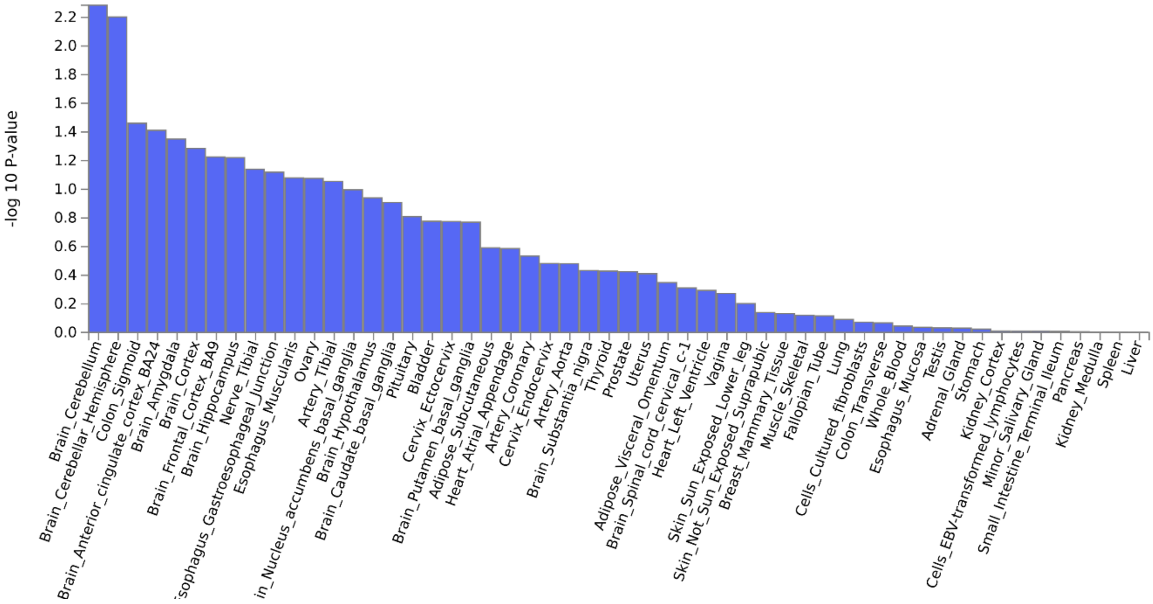

Supplementary Figure 4 – GTEx v8 53 tissue types for language network

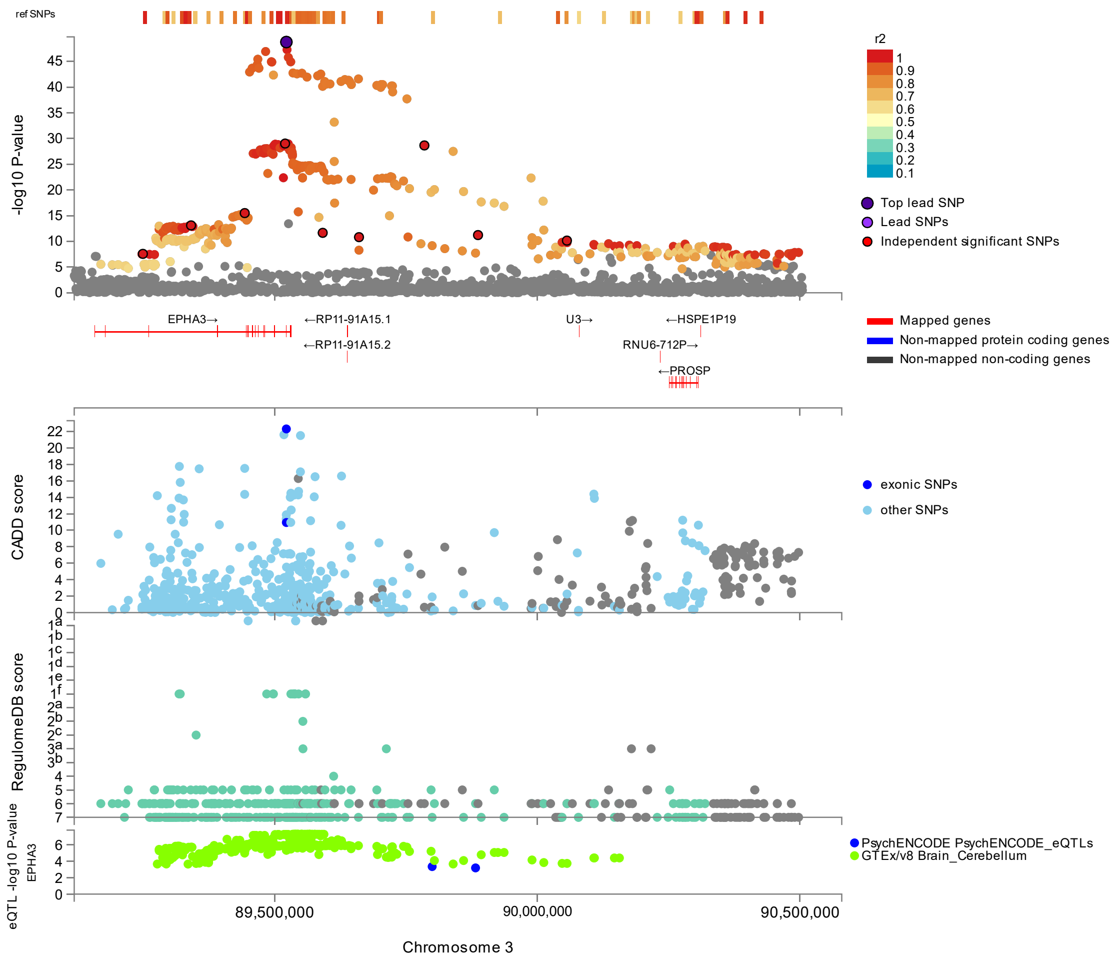

Supplementary Figure 5 – Locuszoom plot for language network results of rs35124509 on chromosome 3. Top: a fine-mapping plot is shown with lead SNPs and linkage disequilibrium (r2). Middle: Combined Annotation Dependent Depletion (CADD) scores are shown, which predict a functional protein effect. Bottom: RegulomeDB scores are shown, which predict interaction effects and gene expression effects using expression quantitative trait loci (eQTL), relating to psychiatric disorders and brain expression.

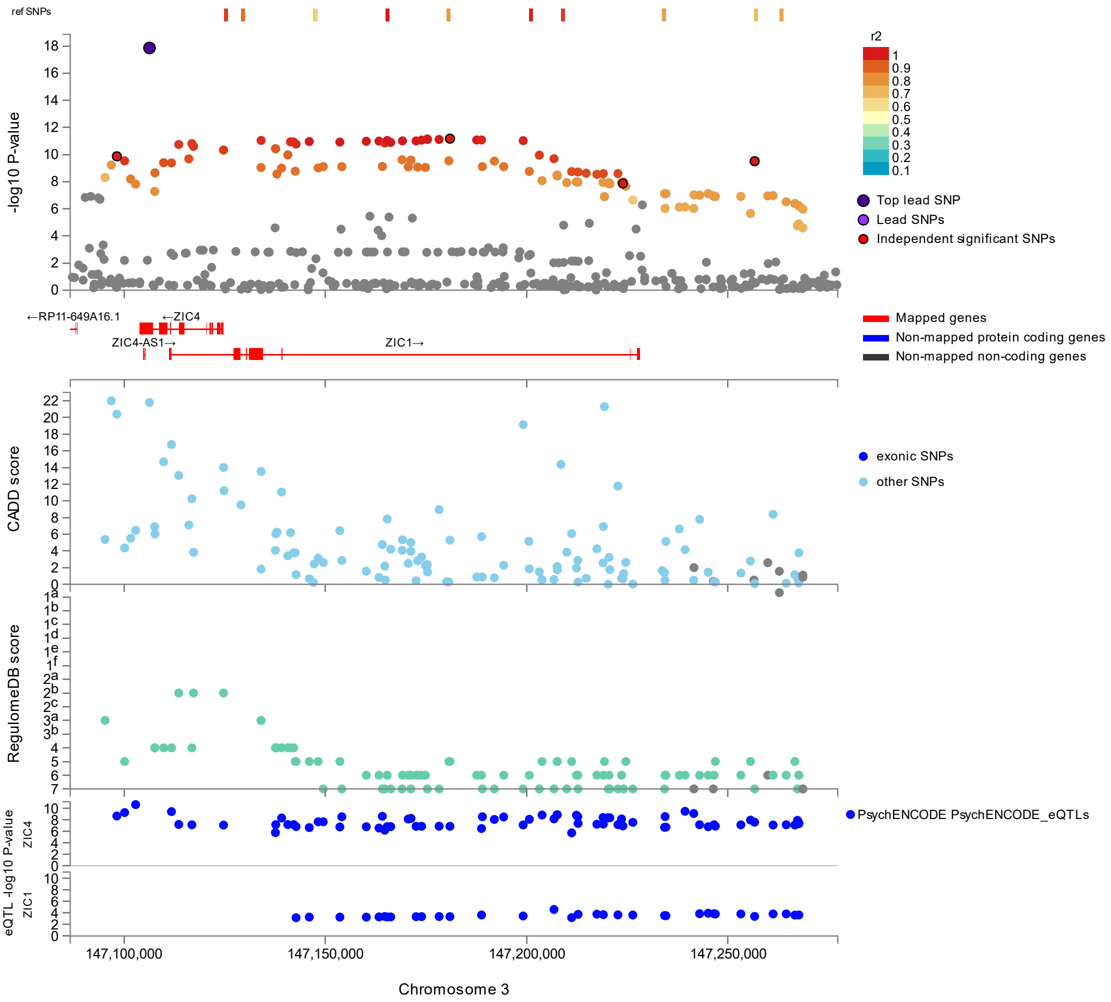

Supplementary Figure 6 – Locuszoom plot for language network results of rs2279829 on chromosome 3. Top: a fine-mapping plot is shown with lead SNPs and linkage disequilibrium (r2). Middle: Combined Annotation Dependent Depletion (CADD) scores are shown, which predict a functional protein effect. Bottom: RegulomeDB scores are shown, which predict interaction effects and gene expression effects using expression quantitative trait loci (eQTL), relating to psychiatric disorders and brain expression.

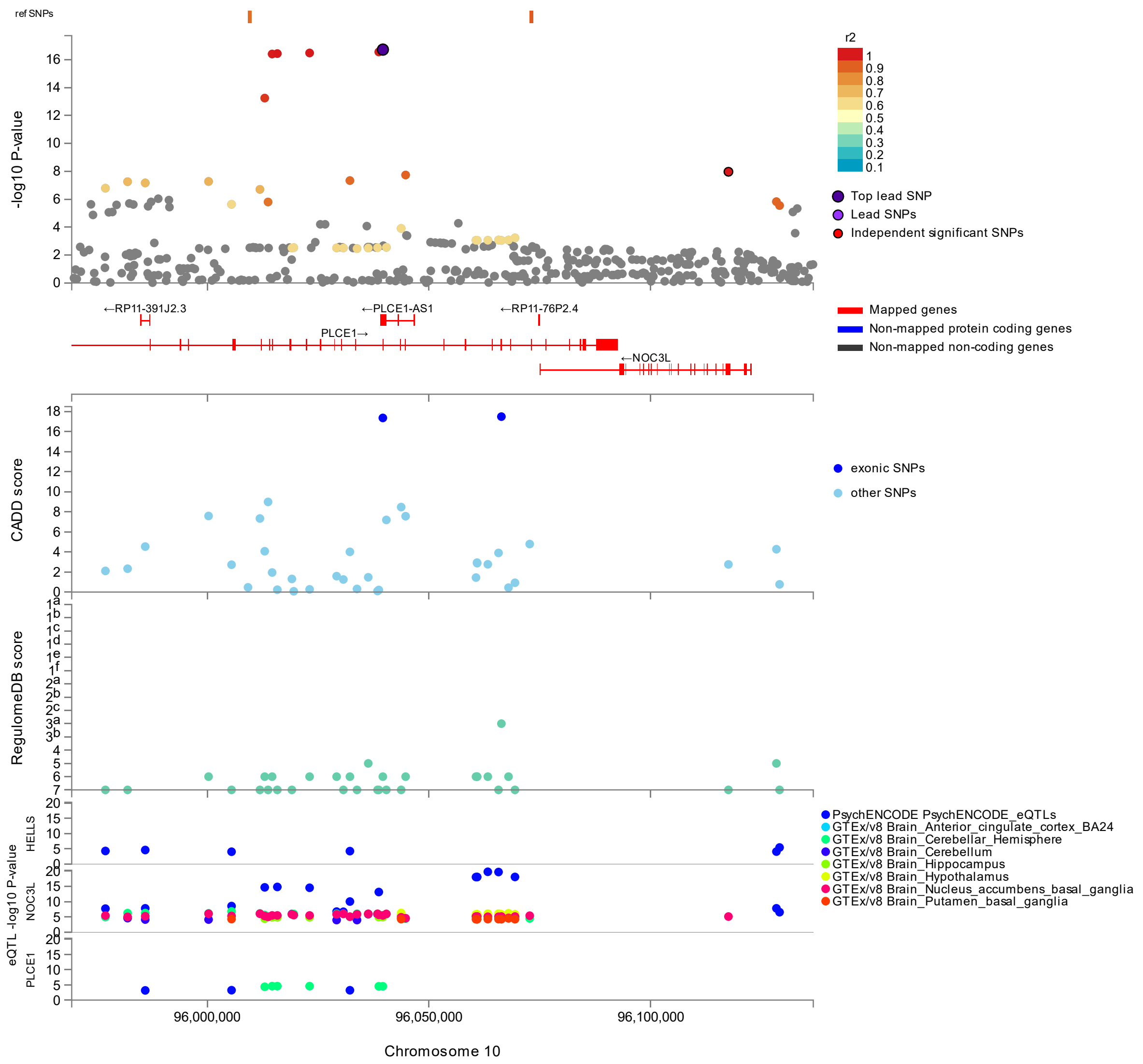

Supplementary Figure 7 - Locuszoom plot for language network results of rs2274224 on chromosome 10. Top: a fine-mapping plot is shown with lead SNPs and linkage disequilibrium (r2). Middle: Combined Annotation Dependent Depletion (CADD) scores are shown, which predict a functional protein effect. Bottom: RegulomeDB scores are shown, which predict interaction effects and gene expression effects using expression quantitative trait loci (eQTL), relating to psychiatric disorders and brain expression.

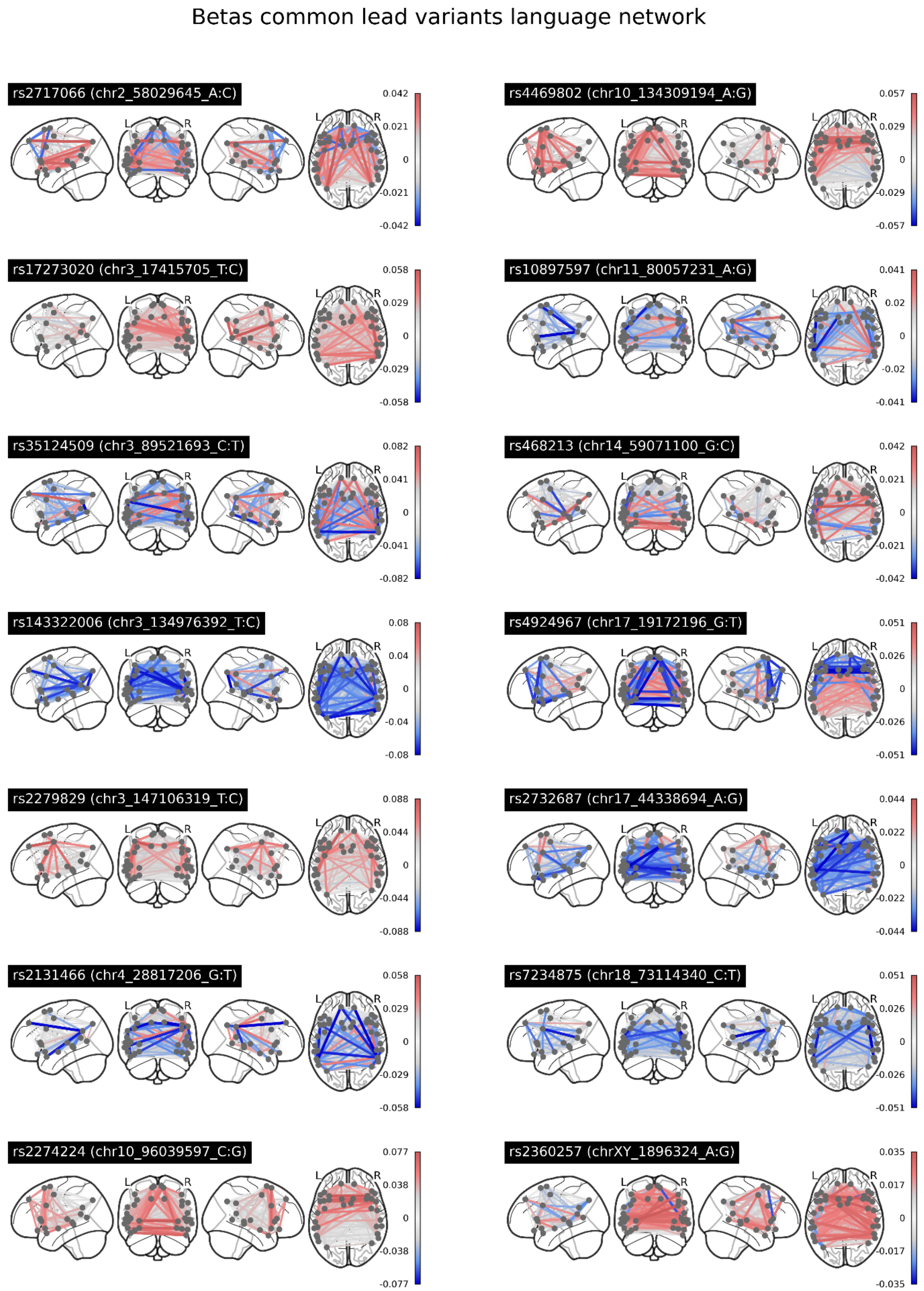

Supplementary Figure 8 - Underlying univariate beta weights for all 14 significant lead SNPs for language network edges. Red indicates a positive association of a given edge or hemispheric difference with increasing number of the minor allele of the genetic variant, and blue indicates a negative association.

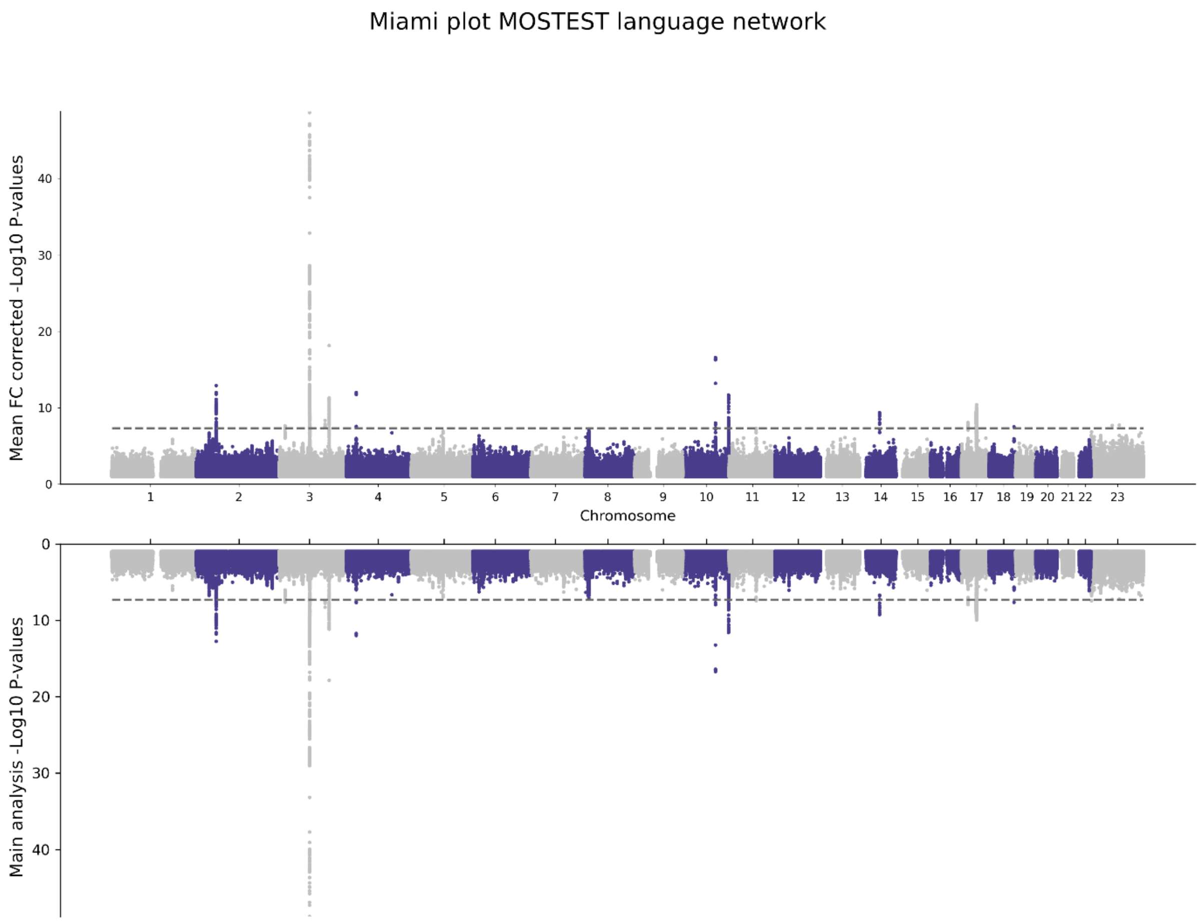

Supplementary Figure 9 – Miami plot for the language network with on top the sensitivity analysis that also corrected for mean functional connectivity and mirrored below the original results. The x-axis shows position on the genome, whereas the y-axis shows -log10 P-value for each association.

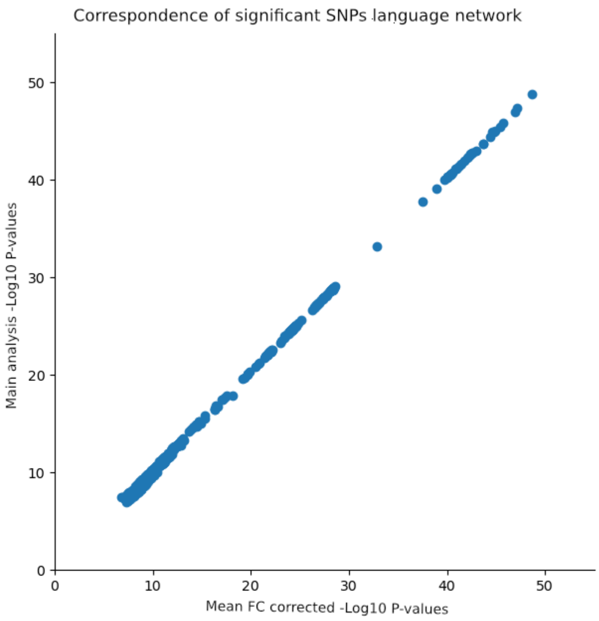

Supplementary Figure 10 – Scatterplot for language network significant SNPs with on the x-axis the sensitivity analysis and on the y-axis the original results.

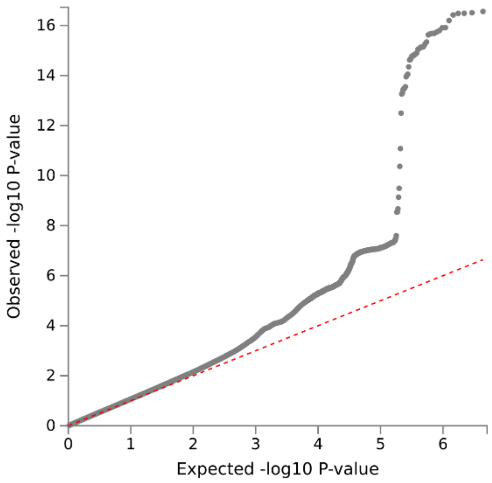

Supplementary Figure 11 – QQ plot for mvGWAS results hemispheric differences

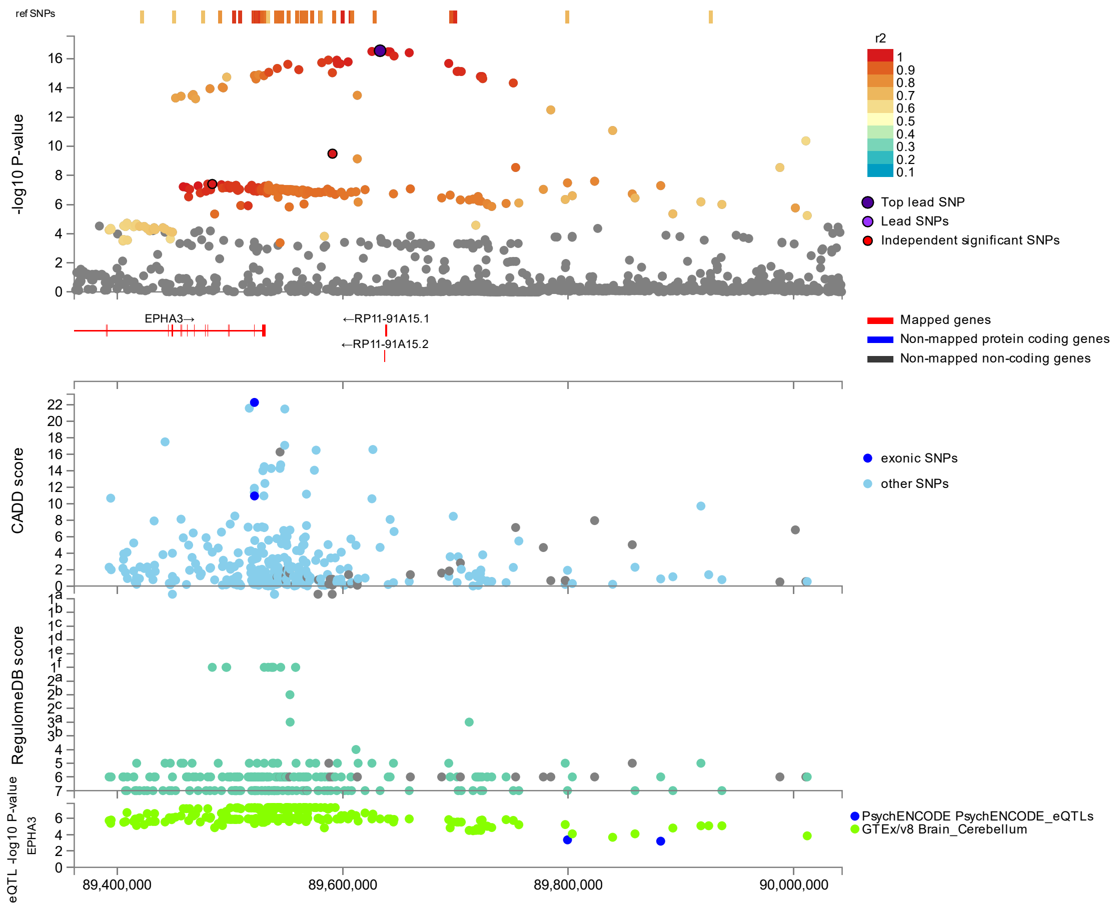

Supplementary Figure 12 – Locuszoom plot for hemispheric differences results of rs7625916 on chromosome 3. Top: a fine-mapping plot is shown with lead SNPs and linkage disequilibrium (r2). Middle: Combined Annotation Dependent Depletion (CADD) scores are shown, which predict a functional protein effect. Bottom: RegulomeDB scores are shown, which predict interaction effects and gene expression effects using expression quantitative trait loci (eQTL), relating to psychiatric disorders and brain expression.

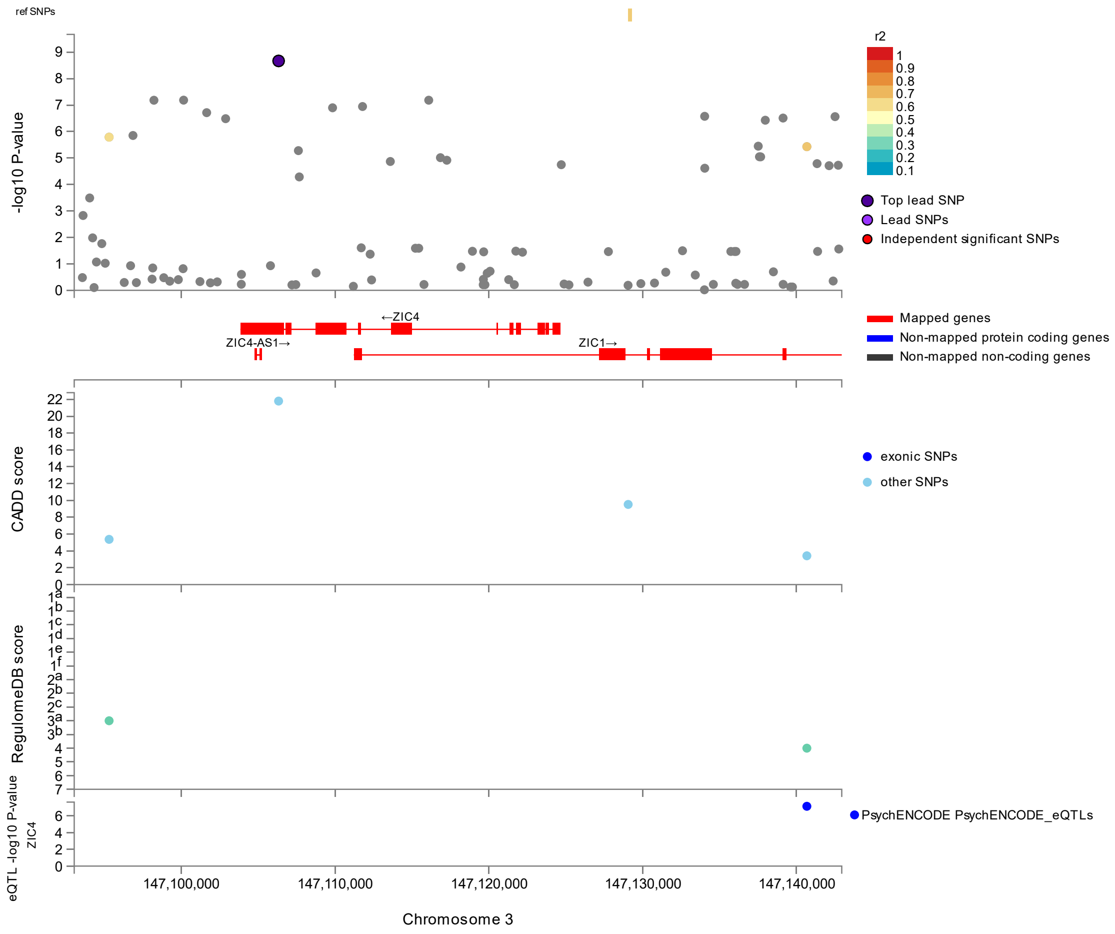

Supplementary Figure 13 – Locuszoom plot for hemispheric differences results of rs2279829 on chromosome 3. Top a fine-mapping plot is shown with lead SNPs and linkage disequilibrium (r2). Middle: Combined Annotation Dependent Depletion (CADD) scores are shown, which predict a functional protein effect. Bottom: RegulomeDB scores are shown, which predict interaction effects and gene expression effects using expression quantitative trait loci (eQTL), relating to psychiatric disorders and brain expression.

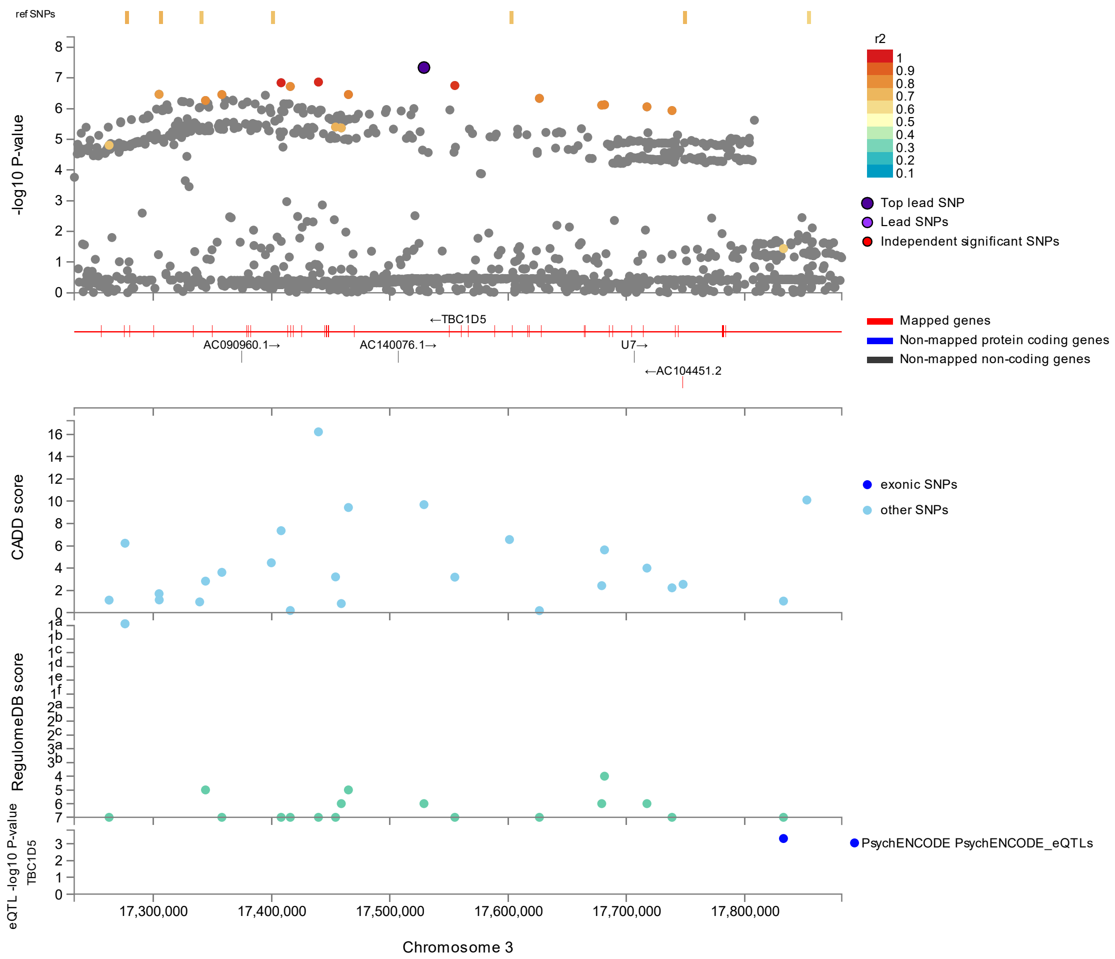

Supplementary Figure 14 – Locuszoom plot for hemispheric differences results of rs1332197 on chromosome 3. Top: a fine-mapping plot is shown with lead SNPs and linkage disequilibrium (r2). Middle: Combined Annotation Dependent Depletion (CADD) scores are shown, which predict a functional protein effect. Bottom: RegulomeDB scores are shown, which predict interaction effects and gene expression effects using expression quantitative trait loci (eQTL), relating to psychiatric disorders and brain expression.

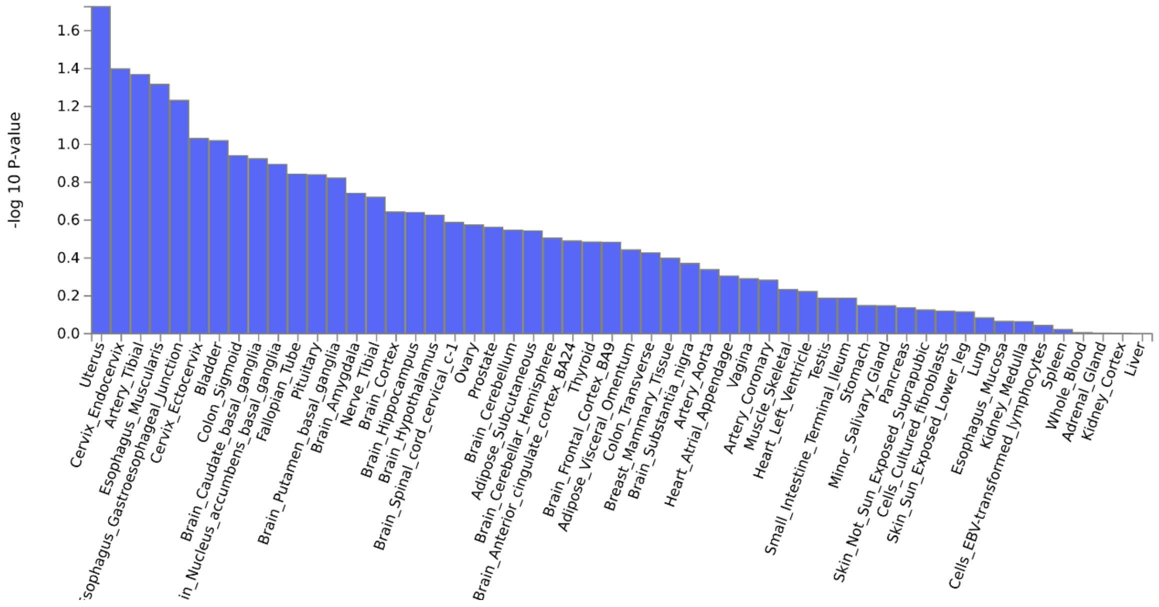

Supplementary Figure 15 - GTEx v8 53 tissue types for hemispheric differences

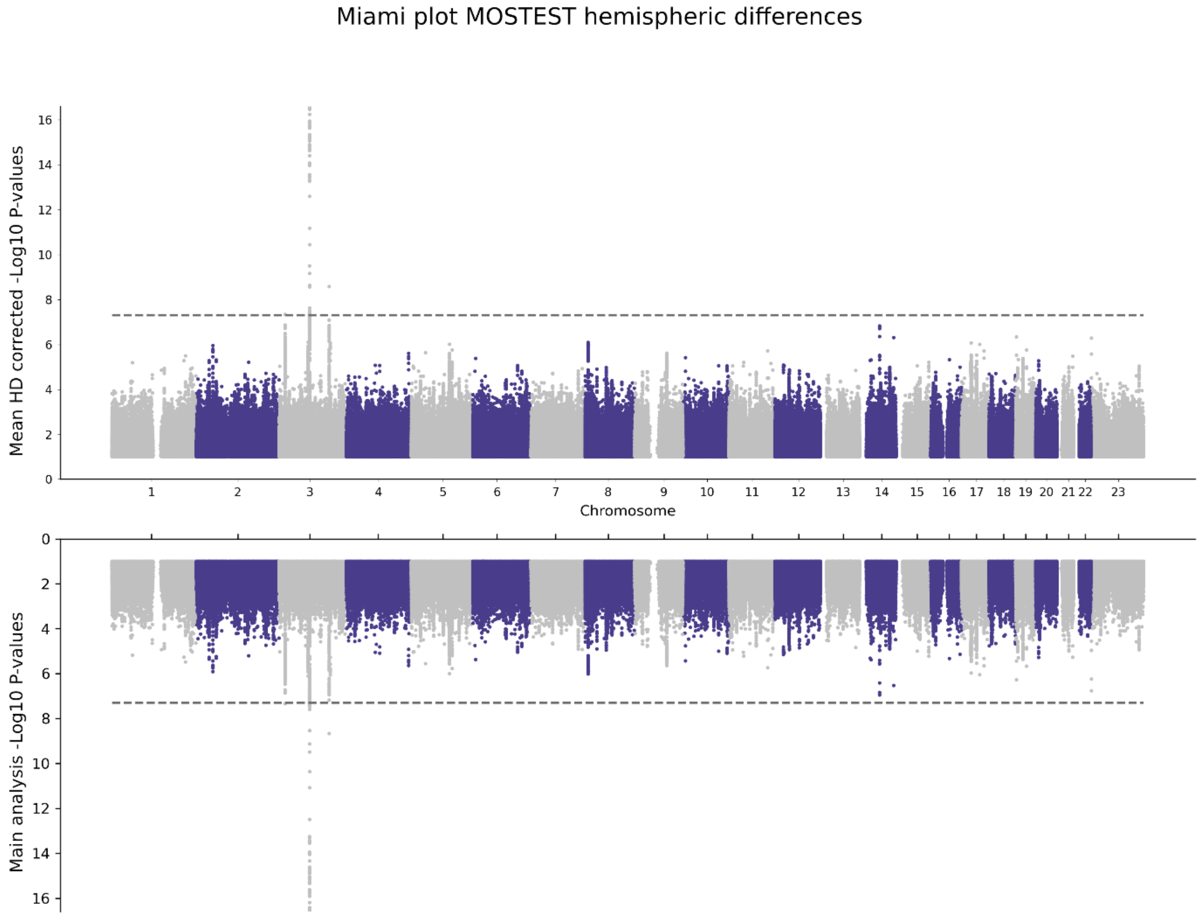

Supplementary Figure 16 - Miami plot for hemispheric differences with on top the sensitivity analysis that also corrected for mean functional connectivity and mirrored below the original results. The x-axis shows position on the genome, whereas the y-axis shows -log10 P-value for each association.

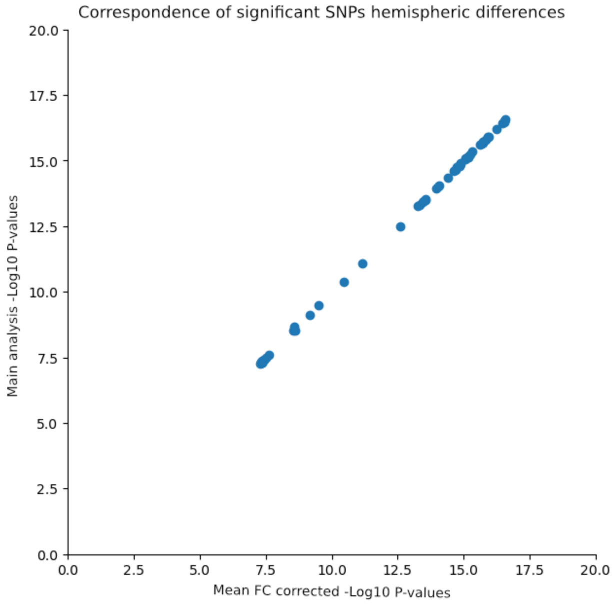

Supplementary Figure 17 – Scatterplot for hemispheric differences significant SNPs with on the x-axis the sensitivity analysis and on the y-axis the original results.

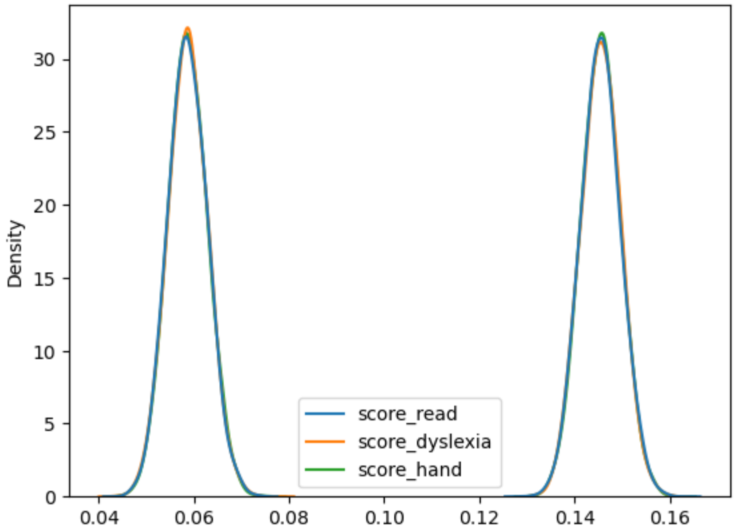

Supplementary Figure 18 – Null distributions for CCA results with permuted language-related abilities, dyslexia and left-handedness polygenic scores. Left distribution is hemispheric differences, right is language network.

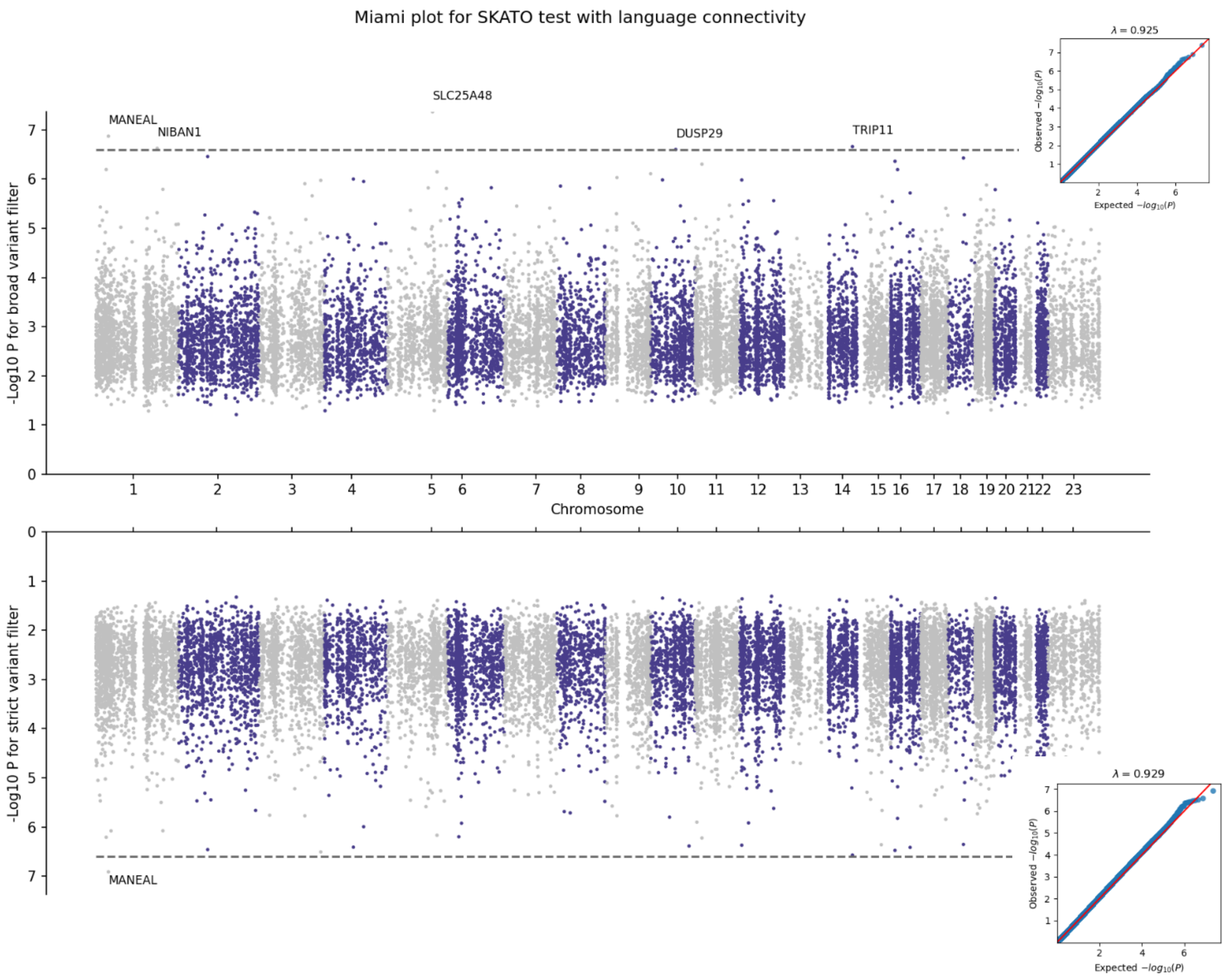

Supplementary Figure 19 – Miami plot for exome-wide gene-based lowest *p*-value associations with language network. Top results are with a broad variant filter, bottom results are with a strict variant filter. QQ plot inserts show genomic inflation for all *p*-values.

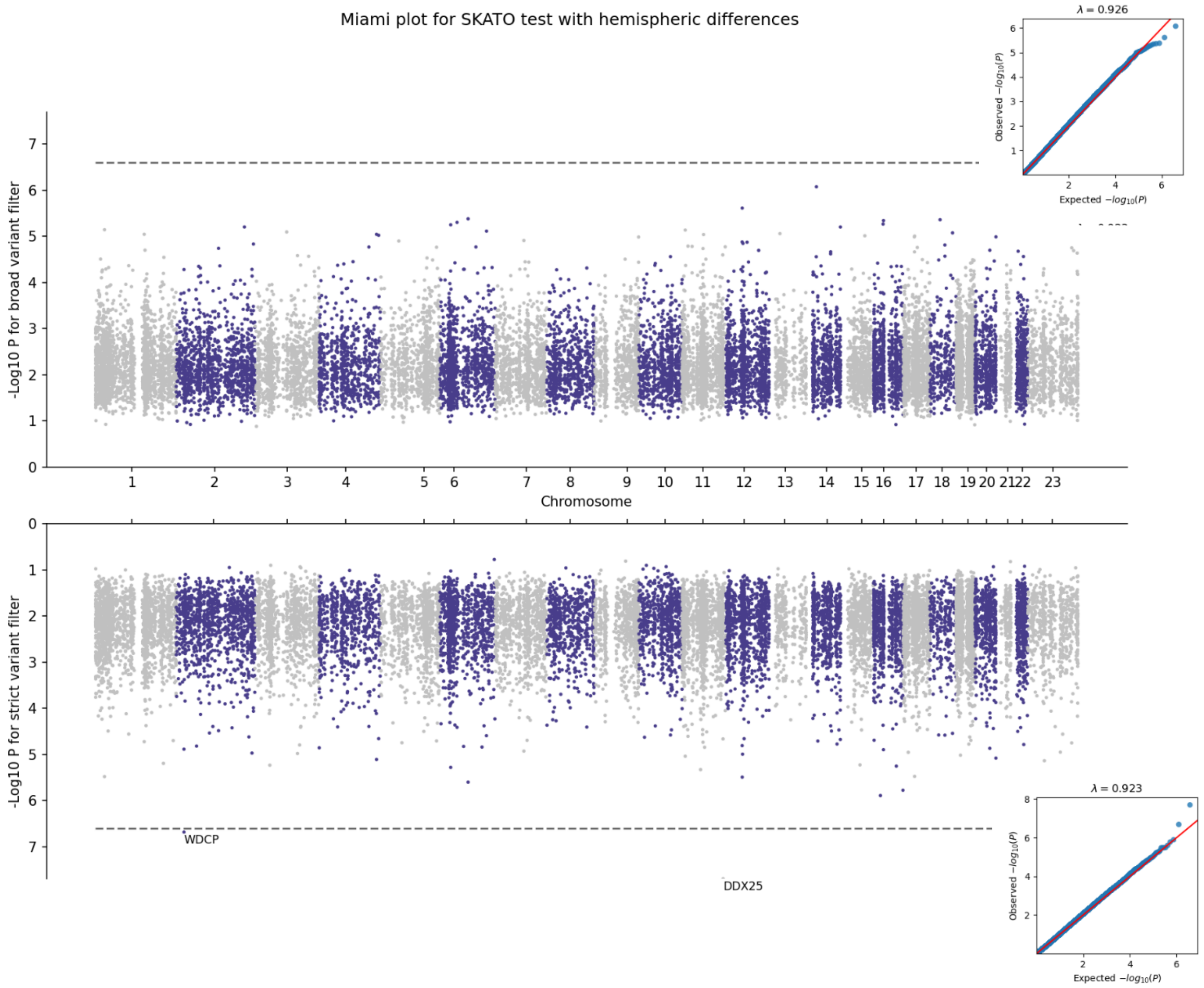

Supplementary Figure 20 - Miami plot for exome-wide gene-based lowest *p*-value associations with hemispheric differences. Top results are with a 'broad' variant filter, bottom results are with a 'strict' variant filter (see Methods). QQ plot inserts show genomic inflation for all *p*-values.

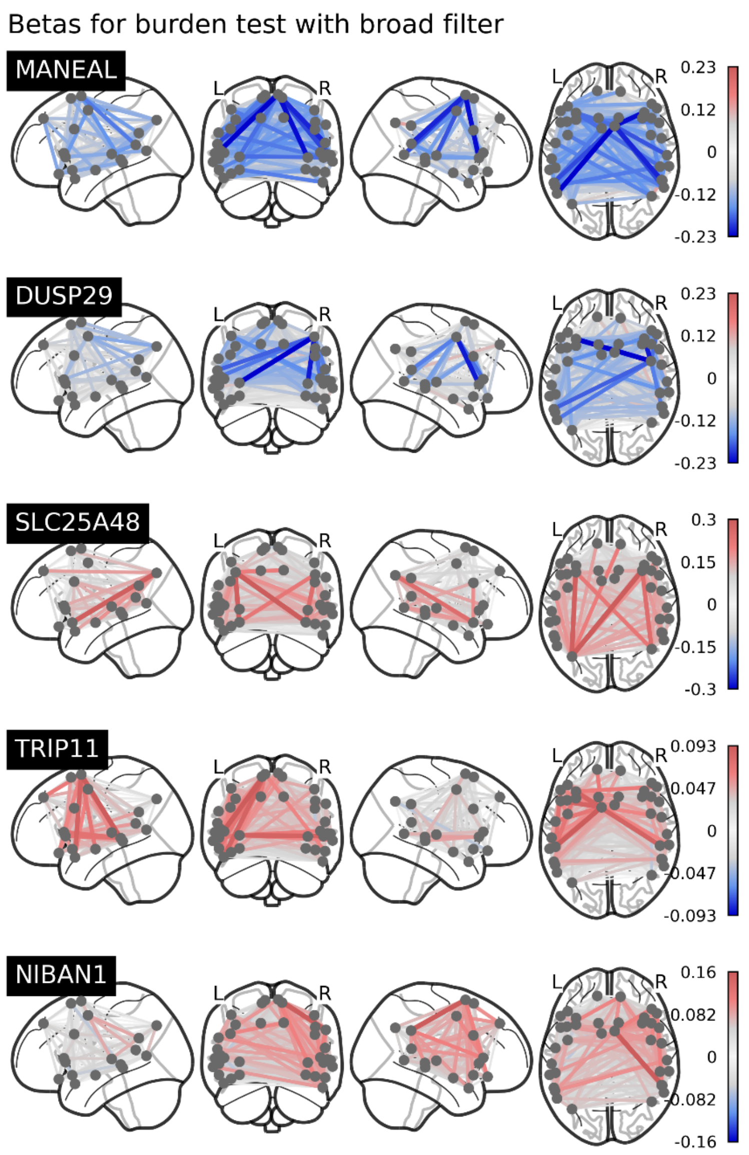

Supplementary Figure 21 – Language network betas for increased genetic burden with a 'broad' variant filter (see Methods). Red means an increase in connectivity, blue means a decrease in connectivity.

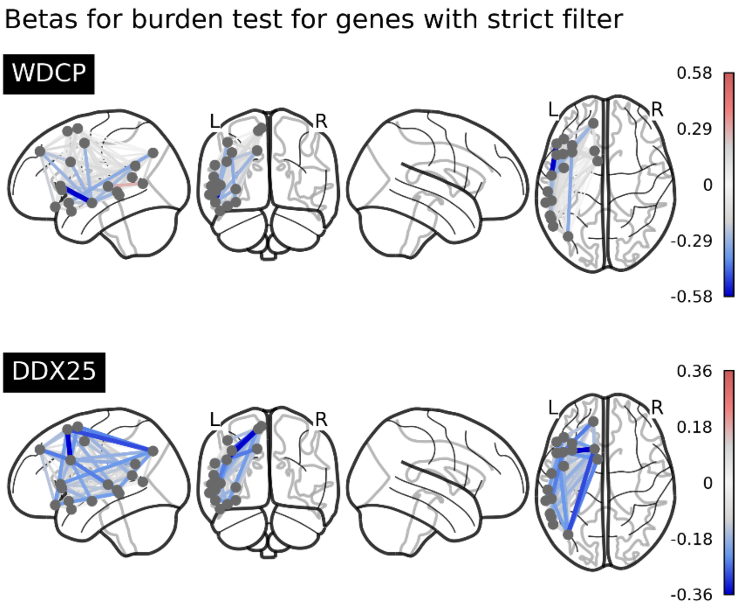

Supplementary Figure 22 – Hemispheric differences betas for increased genetic burden with a ‘strict’ variant filter (see Methods). Red means an increase in connectivity, blue means a decrease in connectivity.

Supplementary Table 1

UK Biobank field IDs for covariates included in this study

Covariate	UKB field
sex	31
age	calculated from 34 and 52 and 53
age^2,	n/a
age*sex	n/a
Genetic principle components 1-10	22009.1-10
genotype array (binary variable)	coded from 22000
scanner X, Y and Z-position	25756-8
inverted temporal signal to noise ratio	25743
mean framewise displacement	25741

Supplementary Table 2

Overview table for exome variant annotation and which filter (broad or strict or exclusion) was applied.

Variant type	Putative impact	Note
chromosome_deletion	HIGH	Strict
chromosome_duplication	HIGH	Strict
chromosome_deletion	HIGH	Strict
exon_loss_variant	HIGH	Strict
exon_duplication	HIGH	Strict
exon_inversion	HIGH	Strict
frameshift_variant	HIGH	Strict
feature_ablation	HIGH	Strict
gene_fusion	HIGH	Strict
gene_fusion	HIGH	Strict
bidirectional_gene_fusion	HIGH	Strict
rearranged_at_DNA_level	HIGH	Strict
protein_protein_contact	HIGH	Strict
structural_interaction_variant	HIGH	Strict
rare_amino_acid_variant	HIGH	Strict
splice_acceptor_variant	HIGH	Strict
splice_donor_variant	HIGH	Strict
stop_lost	HIGH	Strict
start_lost	HIGH	Strict
stop_gained	HIGH	Strict
feature_ablation	HIGH	Strict
inframe_insertion	MODERATE	Strict, Phred>20. Broad, Phred>1.
disruptive_inframe_insertion	MODERATE	Strict, Phred>20. Broad, Phred>1.
inframe_deletion	MODERATE	Strict, Phred>20. Broad, Phred>1.
disruptive_inframe_deletion	MODERATE	Strict, Phred>20. Broad, Phred>1.
missense_variant	MODERATE	Strict, Phred>20. Broad, Phred>1.
splice_region_variant	MODERATE	Strict, Phred>20. Broad, Phred>1.
3_prime_UTR_truncation + exon_loss	MODERATE	Strict, Phred>20. Broad, Phred>1.
5_prime_UTR_truncation + exon_loss_variant	MODERATE	Strict, Phred>20. Broad, Phred>1.
sequence_feature + exon_loss_variant	MODERATE	Strict, Phred>20. Broad, Phred>1.
coding_sequence_variant	LOW	Excluded
initiator_codon_variant	LOW	Excluded
stop_retained_variant	LOW	Excluded
splice_region_variant	LOW	Excluded
splice_region_variant	LOW	Excluded
5_prime_UTR_premature_start_codon_gain_variant	LOW	Excluded
synonymous_variant	LOW	Excluded
start_retained	LOW	Excluded
stop_retained_variant	LOW	Excluded
coding_sequence_variant	MODIFIER	Broad, Phred>1.
downstream_gene_variant	MODIFIER	Broad, Phred>1.

exon_variant	MODIFIER	Broad, Phred>1.
gene_variant	MODIFIER	Broad, Phred>1.
duplication	MODIFIER	Broad, Phred>1.
intergenic_region	MODIFIER	Broad, Phred>1.
conserved_intergenic_variant	MODIFIER	Broad, Phred>1.
intragenic_variant	MODIFIER	Broad, Phred>1.
intron_variant	MODIFIER	Broad, Phred>1.
conserved_intron_variant	MODIFIER	Broad, Phred>1.
miRNA	MODIFIER	Broad, Phred>1.
transcript_variant	MODIFIER	Broad, Phred>1.
regulatory_region_variant	MODIFIER	Broad, Phred>1.
upstream_gene_variant	MODIFIER	Broad, Phred>1.
3_prime_UTR_variant	MODIFIER	Broad, Phred>1.
5_prime_UTR_variant	MODIFIER	Broad, Phred>1.
