# Supplementary material for: Imaging genetics of language network functional connectivity reveals links with language-related abilities, dyslexia and handedness
Source: Commun Biol. 2024 Sep 28;7:1209. doi: 10.1038/s42003-024-06890-3 (PMC11438961; doi:10.1038/s42003-024-06890-3)
Supplement: Supplementary file 2 — Description of Additional Supplementary Materials [file 42003_2024_6890_MOESM2_ESM.pdf]

# Description of Additional Supplementary Files

**File name:** Supplementary Data 1

**Description:** Heritability estimates and significance indications for all 783 imaging derived phenotypes: language network (categories L, R and inter) and hemispheric differences (edge HD).

**File name:** Supplementary Data 2

**Description:** An overview of all 14 loci discovered in a multivariate GWAS of the language network

**File name:** Supplementary Data 3

**Description:** 111 Mapped genes by FUMA based on multivariate GWAS results of the language network

**File name:** Supplementary Data 4

**Description:** Gene set preferential brain expression in Brainspan dataset for language network genes as used in figure 1C. Derived using MAGMA.

**File name:** Supplementary Data 5

**Description:** Gene set analysis of the language network results with MAGMA as implemented in FUMA. Gene sets smaller than 10 were excluded.

**File name:** Supplementary Data 6

**Description:** An overview of all underlying univariate beta weights for the lead SNPs of 14 loci discovered in a multivariate GWAS of the language network used in figure 1E and Supplementary Figure 8

**File name:** Supplementary Data 7

**Description:** Underlying univariate P-values of lead SNPs for each significant language network genomic locus

**File name:** Supplementary Data 8

**Description:** T-tests for comparison of global change in language network connectivity, left and right intrahemispheric connectivity, and intra and interhemispheric connectivity for 14 significant genomic loci

**File name:** Supplementary Data 9

**Description:** An overview of all 3 loci discovered in a multivariate GWAS of hemispheric differences of the language network

**File name:** Supplementary Data 10

**Description:** 9 Mapped genes by FUMA based on multivariate GWAS results of hemispheric differences in the language network

**File name:** Supplementary Data 11

**Description:** Gene set preferential brain expression in Brainspan dataset for language network hemispheric differences genes as used in figure 1D. Derived using MAGMA.

**File name:** Supplementary Data 12

**Description:** Gene set analysis of the hemispheric differences results with MAGMA as implemented in FUMA. Gene sets smaller than 10 were excluded.

**File name:** Supplementary Data 13

**Description:** An overview of all underlying univariate beta weights for the lead SNPs of 3 loci discovered in a multivariate GWAS of hemispheric differences in the language network used in figure 1F

**File name:** Supplementary Data 14

**Description:** An overview of all underlying univariate P-values for the lead SNPs of 3 loci discovered in a multivariate GWAS of hemispheric differences in the language network

**File name:** Supplementary Data 15

**Description:** T-test results for global left-right shift in hemispheric differences for 3 significant genomic loci

**File name:** Supplementary Data 16

**Description:** CCA loadings of individual IDPs for language network with polygenic scores used in figure 2A.

**File name:** Supplementary Data 17

**Description:** T-tests for comparison of global change in language network connectivity, left and right intrahemispheric connectivity, and intra and interhemispheric connectivity for 3 polygenic scores

**File name:** Supplementary Data 18

**Description:** CCA loadings of individual IDPs for hemispheric differences with polygenic scores used in figure 2B.

**File name:** Supplementary Data 19

**Description:** T-test results for global left-right shift in hemispheric differences for 3 polygenic scores

**File name:** Supplementary Data 20

**Description:** Gene-based associations (-log10 p-value) with the language network (MINP_edges) and hemispheric differences (MINP_HDs) when applying a broad variant filter.Average (avg_LOGP) and lowest -log10 p-values (MINP) are reported, as well as the univariate association driving the result. GENPOS refers to position according to positions in genome build GRCh38.

**File name:** Supplementary Data 21

**Description:** Broad variant filter gene-based SKATO associations (-log10 p-value) for all edges of the language network as shown in figure 3A and 3C

**File name:** Supplementary Data 22

**Description:** Gene-based associations (-log10 p-value) with the language network (MINP_edges) and hemispheric differences (MINP_HDs) when applying a strict variant filter. Average (avg_LOGP) and

lowest -log10 p-values (MINP) are reported, as well as the univariate association driving the result. GENPOS refers to position according to positions in GRCh38.

**File name:** Supplementary Data 23

**Description:** Strict variant filter gene-based SKATO associations (-log10 p-value) for all edges of the language network hemispheric differences as shown in figure 3B and 3D

**File name:** Supplementary Data 24

**Description:** Variant-based associations (-log10 P values) with the language network (MINP_edges) and hemispheric differences (MINP_HDs) for the exome-wide significant genes.

**File name:** Supplementary Data 25

**Description:** Univariate z-statistics from a gene-based burden test (broad filter) for all 629 heritable imaging derived phenotypes of the language network

**File name:** Supplementary Data 26

**Description:** T-tests for comparison of global change in language network connectivity, left and right intrahemispheric connectivity, and intra and interhemispheric connectivity for 5 significant genes

**File name:** Supplementary Data 27

**Description:** Univariate z-statistics from a gene-based burden test (strict filter) for all 103 heritable imaging derived phenotypes of hemispheric differences in the language network

**File name:** Supplementary Data 28

**Description:** T-test results for global left-right shift in hemispheric difference for 2 significant genes

**File name:** Supplementary Data 29

**Description:** Brainspan RNA-seq RKPM expression levels for significant genes in rare variant analysis that are available in the rare variant analysis used in figure 3E
