# Supplementary material for: Imaging genetics of language network functional connectivity reveals links with language-related abilities, dyslexia and handedness
Source: Commun Biol. 2024 Sep 28;7:1209. doi: 10.1038/s42003-024-06890-3 (PMC11438961; doi:10.1038/s42003-024-06890-3)
Supplement: Supplementary file 4 — Reporting Summary [file 42003_2024_6890_MOESM4_ESM.pdf]

# nature portfolio

Corresponding author(s): Clyde Francks

Last updated by author(s): Aug 30, 2024

# Reporting Summary

Nature Portfolio wishes to improve the reproducibility of the work that we publish. This form provides structure for consistency and transparency in reporting. For further information on Nature Portfolio policies, see our Editorial Policies and the Editorial Policy Checklist.

## Statistics

For all statistical analyses, confirm that the following items are present in the figure legend, table legend, main text, or Methods section.

| n/a | Confirmed | |
|---|---|---|
| ☐ | ☒ | The exact sample size (*n*) for each experimental group/condition, given as a discrete number and unit of measurement |
| ☐ | ☒ | A statement on whether measurements were taken from distinct samples or whether the same sample was measured repeatedly |
| ☐ | ☒ | The statistical test(s) used AND whether they are one- or two-sided<br>*Only common tests should be described solely by name; describe more complex techniques in the Methods section.* |
| ☐ | ☒ | A description of all covariates tested |
| ☐ | ☒ | A description of any assumptions or corrections, such as tests of normality and adjustment for multiple comparisons |
| ☐ | ☒ | A full description of the statistical parameters including central tendency (e.g. means) or other basic estimates (e.g. regression coefficient) AND variation (e.g. standard deviation) or associated estimates of uncertainty (e.g. confidence intervals) |
| ☐ | ☒ | For null hypothesis testing, the test statistic (e.g. *F*, *t*, *r*) with confidence intervals, effect sizes, degrees of freedom and *P* value noted<br>*Give P values as exact values whenever suitable.* |
| ☒ | ☐ | For Bayesian analysis, information on the choice of priors and Markov chain Monte Carlo settings |
| ☒ | ☐ | For hierarchical and complex designs, identification of the appropriate level for tests and full reporting of outcomes |
| ☐ | ☒ | Estimates of effect sizes (e.g. Cohen's *d*, Pearson's *r*), indicating how they were calculated |

*Our web collection on statistics for biologists contains articles on many of the points above.*

## Software and code

Policy information about availability of computer code

| | |
|---|---|
| Data collection | Data pre-processing was previously performed by the UK Biobank (not by us specifically for this study) and we cite their protocols in the Methods section.<br>For the present study, we extracted resting-state functional image time courses according to the AICHA atlas using invwarp and applywarp from FSL (v. 5.0.10) and mri segstats from Freesurfer (v.6.0.0 ). Correlations between time courses were derived with numpy (v.1.13.1) using Python 2.7. Custom code for this study is available from https://github.com/jsamelink/langnet_paper |

| Data analysis | Heritability analyses were performed in GCTA v. 1.93.0beta.<br>Multivariate common variant association testing (mvGWAS) was performed using the MOSTest toolbox (no version number given with the release).<br>Genome-wide significant variants were annotated using FUMA (version 1.5.2).<br>MAGMA (version 1.08) implemented within FUMA was used to calculate gene-based p-values and for gene-property analyses, to investigate potential gene sets of interest and to map the expression of associated genes in a tissue-specific and time-specific fashion.<br>Polygenic scores were calculated with PRS-CS (Nov 2022 version).<br>Polygenic scores were then normalized using quantile transform from scikit-learn v.1.0.1 and entered into canonical correlation analysis with respect to brain functional connectivity measures, also using scikit-learn.<br>For rare variant association testing REGENIE v.3.2.1 was used.<br>CADD Phred scores for variants were taken from the database for nonsynonymous functional prediction (dbNSFP) (version 4.3a) using snpSift 5.1d(build 2022-04-19).<br>Functional annotation of variants was conducted using snpEff v5.1d (build 2022-04-19). |
|---|---|

For manuscripts utilizing custom algorithms or software that are central to the research but not yet described in published literature, software must be made available to editors and reviewers. We strongly encourage code deposition in a community repository (e.g. GitHub). See the Nature Portfolio guidelines for submitting code & software for further information.

## Data

Policy information about availability of data

All manuscripts must include a data availability statement. This statement should provide the following information, where applicable:

- Accession codes, unique identifiers, or web links for publicly available datasets
- A description of any restrictions on data availability
- For clinical datasets or third party data, please ensure that the statement adheres to our policy

The primary data used in this study are from the UK Biobank. These data can be provided by UK Biobank pending scientific review and a completed material transfer agreement. Requests for the data should be submitted to the UK Biobank: https://www.ukbiobank.ac.uk. Specific UK Biobank data field codes are given in Materials and Methods. Other publicly available data sources and applications are cited in Materials and Methods. We have made our mvGWAS summary statistics available online within the GWAS catalog: https://ebi.ac.uk/gwas/. This study  used openly available software and codes, specifically GCTA (https://cnsgenomics.com/software/gcta/#GREML), MOSTest (https://github.com/precimed/mostest), FUMA (https://fuma.ctglab.nl/), MAGMA (https://ctg.cncr.nl/software/magma, also implemented in FUMA), PRS-CS (https://github.com/getian107/PRScs), REGENIE (https://rgcgithub.github.io/regenie/install/) and LD score regression (https://github.com/bulik/ldsc). Custom code for this study is available from https://github.com/jsamelink/langnet_paper. All other data needed to evaluate the conclusions in the paper are present in the paper and/or the Supplementary Materials.

## Research involving human participants, their data, or biological material

Policy information about studies with human participants or human data. See also policy information about sex, gender (identity/presentation), and sexual orientation and race, ethnicity and racism.

| Reporting on sex and gender | We refer to sex as biological attribute. Sex was included as a covariate effect in the analyses. Individuals with discrepancies between their self-reported sex and their genetically determined sex were excluded from the analyses as this can indicate error in data entry. |
|---|---|
| Reporting on race, ethnicity, or other socially relevant groupings | To avoid confounding our genetic association analysis, we restricted the analysis to the single largest group in terms of genetic ancestry, i.e. those individuals who reported having white British ancestry and were found to cluster togetheraccording to principal component analysis of genetic variant data. |
| Population characteristics | 29,681 participants from the UK Biobank between ages 45 and 82 years. |
| Recruitment | We cite the relevant UK Biobank publications as they recruited the individuals (no recruitment was done for our study). |
| Ethics oversight | National Research Ethics Service Committee North West-Haydock (reference 11/NW/0382) |

Note that full information on the approval of the study protocol must also be provided in the manuscript.

# Field-specific reporting

Please select the one below that is the best fit for your research. If you are not sure, read the appropriate sections before making your selection.

☒ Life sciences ☐ Behavioural & social sciences ☐ Ecological, evolutionary & environmental sciences

For a reference copy of the document with all sections, see nature.com/documents/nr-reporting-summary-flat.pdf

# Life sciences study design

All studies must disclose on these points even when the disclosure is negative.

| Sample size | No statistical method was used to predetermine the sample size. It could not be estimated in advance what type of genetic effects on langugue |
|---|---|

| | |
|---|---|
| Sample size | network connectivity would be present in the data. We therefore used the maximum available sample size to maximise the available statistical power to screen the genome for such effects.<br>The sample therefore included all participants from the UK Biobank who met a set of criteria according to data availability, quality, and population genetic ancestry, as detailed in the Methods section and repeated here below. The multi-step process through which we arrived at the study's final sample size is most correctly and accurately described by pasting from the Methods section, because all of this information determined the final sample size:<br>Sample-level quality control at the phenotypic and genetic level was conducted on 40,595 participants who had imaging, genotype and exome data available. In phenotype sample-level quality control, participants were first excluded with imaging data labelled as unusable by UK Biobank quality control. Second, participants were removed based on outliers (here defined as 6 × interquartile range (IQR)) in at least one of the following metrics: discrepancy between rs-fMRI brain image and T1 structural brain image (UK Biobank field 25739), inverted temporal signal-to-noise ratio in preprocessed and artefact-cleaned preprocessed rs-fMRI (data fields 25743 and 25744), scanner X, Y and Z brain position (fields 25756, 25757 and 25758) or in functional connectivity asymmetries (see section* Imaging data preprocessing and phenotype derivation). Third, participants with missing data in the connectivity matrices were excluded. In total 3,472 participants were excluded in the phenotype QC.<br>Subsequently, in genetic sample-level quality control, only participants in the pre-defined white British ancestry cluster were included (data-field 22006) [34], as this was the largest single cluster in terms of ancestral homogeneity – an important consideration for some of the genetic analyses that we carried out (below). Furthermore, participants were excluded when self-reported sex (data-field 31) did not match genetically inferred sex based on genotype data (data field 22001) or exome data, when sex chromosome aneuploidy was suspected (data-field 22019), or when exclusion thresholds were exceeded in heterozygosity (≥ 0.1903) and/or genotype missingness rate (≥ 0.05) (data-field 22027). Finally, one random member of each pair of related participants (up to third degree, kinship coefficient ≥ 0.0442, pre-calculated by UK Biobank) was removed from the analysis. This led to the further exclusion of 7,442 participants.<br>In total 29,681 participants were included in all further analyses. |
| Data exclusions | The exclusion criteria were pre-established. There were multiple exclusion criteria based on the availability and quality of the different types of data, as well as population genetic homogeneity. The exclusion process is most correctly and accurately described by pasting from the Methods section, as all of these steps are relevant to which data were excluded:<br>Sample-level quality control at the phenotypic and genetic level was conducted on 40,595 participants who had imaging, genotype and exome data available. In phenotype sample-level quality control, participants were first excluded with imaging data labelled as unusable by UK Biobank quality control. Second, participants were removed based on outliers (here defined as 6 × interquartile range (IQR)) in at least one of the following metrics: discrepancy between rs-fMRI brain image and T1 structural brain image (UK Biobank field 25739), inverted temporal signal-to-noise ratio in preprocessed and artefact-cleaned preprocessed rs-fMRI (data fields 25743 and 25744), scanner X, Y and Z brain position (fields 25756, 25757 and 25758) or in functional connectivity asymmetries (see section* Imaging data preprocessing and phenotype derivation). Third, participants with missing data in the connectivity matrices were excluded. In total 3,472 participants were excluded in the phenotype QC.<br>Subsequently, in genetic sample-level quality control, only participants in the pre-defined white British ancestry cluster were included (data-field 22006) [34], as this was the largest single cluster in terms of ancestral homogeneity – an important consideration for some of the genetic analyses that we carried out (below). Furthermore, participants were excluded when self-reported sex (data-field 31) did not match genetically inferred sex based on genotype data (data field 22001) or exome data, when sex chromosome aneuploidy was suspected (data-field 22019), or when exclusion thresholds were exceeded in heterozygosity (≥ 0.1903) and/or genotype missingness rate (≥ 0.05) (data-field 22027). Finally, one random member of each pair of related participants (up to third degree, kinship coefficient ≥ 0.0442, pre-calculated by UK Biobank) was removed from the analysis. This led to the further exclusion of 7,442 participants.<br>In total 29,681 participants were included in all further analyses. |
| Replication | We are not aware of a suitable replication sample to match the scale of the UK Biobank. Thousands of adult indivduals with brain image data, common variant genotyping, and exome sequence data would be required. |
| Randomization | This was not a feature of our observational study. |
| Blinding | This was not a feature of our observational study. |

# Reporting for specific materials, systems and methods

We require information from authors about some types of materials, experimental systems and methods used in many studies. Here, indicate whether each material, system or method listed is relevant to your study. If you are not sure if a list item applies to your research, read the appropriate section before selecting a response.

## Materials & experimental systems

| n/a | Involved in the study | |
|---|---|---|
| ☒ | ☐ | Antibodies |
| ☒ | ☐ | Eukaryotic cell lines |
| ☒ | ☐ | Palaeontology and archaeology |
| ☒ | ☐ | Animals and other organisms |
| ☒ | ☐ | Clinical data |
| ☒ | ☐ | Dual use research of concern |
| ☒ | ☐ | Plants |

## Methods

| n/a | Involved in the study | |
|---|---|---|
| ☒ | ☐ | ChIP-seq |
| ☒ | ☐ | Flow cytometry |
| ☐ | ☒ | MRI-based neuroimaging |

## Plants

| | |
|---|---|
| Seed stocks | Not applicable. We indicated above that no plants were involved in this study, but the dynamic pdf form has included this section anyway. |
| Novel plant genotypes | Not applicable. We indicated above that no plants were involved in this study, but the dynamic pdf form has included this section anyway. |
| Authentication | Not applicable. We indicated above that no plants were involved in this study, but the dynamic pdf form has included this section anyway. |

## Magnetic resonance imaging

### Experimental design

| | |
|---|---|
| Design type | The data were previously obtained by the UK Biobank and the protocol has been described previously. We cite the relevant papers in our manuscript. |
| Design specifications | The data were previously obtained by the UK Biobank and the protocol has been described previously. We cite the relevant papers in our manuscript. |
| Behavioral performance measures | N/A |

### Acquisition

| | |
|---|---|
| Imaging type(s) | The data were previously obtained by the UK Biobank and the protocol has been described previously. We cite the relevant papers in our manuscript. |
| Field strength | The data were previously obtained by the UK Biobank and the protocol has been described previously. We cite the relevant papers in our manuscript. |
| Sequence & imaging parameters | The data were previously obtained by the UK Biobank and the protocol has been described previously. We cite the relevant papers in our manuscript. |
| Area of acquisition | The data were previously obtained by the UK Biobank and the protocol has been described previously. We cite the relevant papers in our manuscript. |

Diffusion MRI ☐ Used ☒ Not used

### Preprocessing

| | |
|---|---|
| Preprocessing software | These steps were previously performed by the UK Biobank and the protocol has been described previously. We cite the relevant papers in our manuscript. |
| Normalization | These steps were previously performed by the UK Biobank and the protocol has been described previously. We cite the relevant papers in our manuscript. |
| Normalization template | These steps were previously performed by the UK Biobank and the protocol has been described previously. We cite the relevant papers in our manuscript. |
| Noise and artifact removal | These steps were previously performed by the UK Biobank and the protocol has been described previously. We cite the relevant papers in our manuscript. |
| Volume censoring | These steps were previously performed by the UK Biobank and the protocol has been described previously. We cite the relevant papers in our manuscript. |

### Statistical modeling & inference

| | |
|---|---|
| Model type and settings | MOSTEST (multivariate genetic analysis) association analysis of common genetic variants in relation to multiple measures of brain connectivity. MOSTest fully accounts for the multivariate nature by estimating the correlation structure on permuted genotype data and then computing the Mahalanobis norm as the sum of squared de-correlated z-values across univariate associaiton summary statistics and then fitting a null distribution using a gamma cumulative density function to extrapolate beyond the permuted data to significant findings.<br>Canonical correlation analysis (multivariate analysis) of polygenic scores in relation to multiple measures of brain connectivity.<br>Gene-based association testing (univariate) for rare protein-coding variants in relation to single measures of brain |

connectivity.

Effect(s) tested

MOSTEST (multivariate genetic analysis) association analysis of common genetic variants in relation to multiple measures of brain connectivity. MOSTest fully accounts for the multivariate nature by estimating the correlation structure on permuted genotype data and then computing the Mahalanobis norm as the sum of squared de-correlated z-values across univariate associaiton summary statistics and then fitting a null distribution using a gamma cumulative density function to extrapolate beyond the permuted data to significant findings.
Canonical correlation analysis (multivariate analysis) of polygenic scores in relation to multiple measures of brain connectivity.
Gene-based association testing (univariate) for rare protein-coding variants in relation to single measures of brain connectivity.

Specify type of analysis: ☐ Whole brain ☒ ROI-based ☐ Both

Anatomical location(s)

Network connectivity was derived based on the AICHA atlas [reference 30 in the manuscript]. Key properties of the AICHA atlas are its homotopies. For each of the 192 parcels left and right hemisphere functional homotopies were defined. Previous work identified 18 regions as part of the core language network in multiple language processing domains (reading, listening and speaking [reference 3 in the manuscript]). These 18 regions and their homotopies were used in this analysis.

Statistic type for inference

(See Eklund et al. 2016)

MOSTEST (multivariate genetic analysis) association analysis of common genetic variants in relation to multiple measures of brain connectivity. MOSTest fully accounts for the multivariate nature by estimating the correlation structure on permuted genotype data and then computing the Mahalanobis norm as the sum of squared de-correlated z-values across univariate associaiton summary statistics and then fitting a null distribution using a gamma cumulative density function to extrapolate beyond the permuted data to significant findings.
Canonical correlation analysis (multivariate analysis) of polygenic scores in relation to multiple measures of brain connectivity.
Gene-based association testing (univariate) for rare protein-coding variants in relation to single measures of brain connectivity.

Correction

For the multivariate genome-wide associaition analysis we applied the field standard genome-wide multiple testing correction threshold 5x10-8 which has been shown to account for testing millions of single nucleotide polymorphisms with patterns of linkage disequilibrium as found in European ancestry populations.
As correlation values in CCA tend to increase with the number of variables, we permuted the polygenic scores 10,000 times to build a null distribution of correlation values between image-derived measures and permuted polygenic scores and tested whether the correlation values of the first mode were outside the 95th percentile of the null distribution.
We adjusted for the exome-wide gene-based multiple comparison burden using an empirical p-value threshold for Type 1 error control from previous work (2.5 × 10−7 [ reference 33 in the manuscript]). This was computed as 0.05 × the average p-value from 300 random phenotypes with varying heritabilities and UK Biobank exome data and approximates 0.05 expected false positives per phenotype.

## Models & analysis

| n/a | Involved in the study |
|---|---|
| ☐ | ☒ Functional and/or effective connectivity |
| ☒ | ☐ Graph analysis |
| ☒ | ☐ Multivariate modeling or predictive analysis |

Functional and/or effective connectivity

Pearson correlations between time courses were transformed to z-scores using a Fisher transform in order to achieve normality.
